# Neuroretinal and microvascular retinal features in dementia with Lewy body assessed by optical coherence tomography angiography

**DOI:** 10.1007/s10072-024-07683-6

**Published:** 2024-08-17

**Authors:** Giuseppe Maria Albanese, Magda Gharbiya, Giacomo Visioli, Massimiliano Panigutti, Andrea Margarella, Enrico Romano, Elvia Mastrogiuseppe, Micaela Sepe-Monti, Giuseppe Bruno, Fabrizia D’Antonio

**Affiliations:** 1 Department of Sense Organs, Sapienza University of Rome, 155, Viale del Policlinico, Rome, 00161 Italy; 2 Department of Human Neurosciences, Sapienza University of Rome, Rome, 00185 Italy; 3 Department of Clinical Medicine, Public health, Life and Environmental Sciences, University of L’Aquila, L’Aquila, 67010 Italy

**Keywords:** Dementia with Lewy Bodies (DLB), Retinal changes, Optical Coherence Tomography Angiography (OCTA), Retinal Biomarkers, Retinal Nerve Fiber Layer (RNFL), Peripapillary vessel density (ppVD), Vascular Endothelial Growth Factor (VEGF), Neurodegenerative diseases

## Abstract

**Objective:**

To explore retinal changes in patients with Dementia with Lewy Bodies (DLB) using Spectral Domain-Optical Coherence Tomography (SD-OCT) and Optical Coherence Tomography Angiography (OCTA), aiming to identify potential biomarkers for diagnosis and monitoring.

**Methods:**

A cross-sectional study analyzed 15 DLB patients and 18 matched controls. Participants underwent physical, neurological, neuropsychological, and ophthalmological evaluations, including SD-OCT and OCTA. Logistic regression, adjusted for age, sex, and inter-eye correlation, was employed to identify retinal alterations in patients affected by DLB.

**Results:**

OCTA revealed that DLB is associated with reduced superficial and deep vessel densities (SVD and DVD) in the macula (*p* < 0.01), as well as decreased peripapillary vessel density (ppVD, *p* < 0.01). SD-OCT parameters showed correlations with DLB, including reduced central macular thickness (CMT, *p* < 0.001) and thinning of the ganglion cell layer-inner plexiform layer (GCL-IPL, *p* < 0.01). Logistic regression (R²=0.26) identified reduced ppVD as a significant predictor of DLB (*p* = 0.030).

**Conclusions:**

Impairments in retinal capillaries, especially lower ppVD, might mirror cerebral hypoperfusion in DLB, potentially due to reduced Vascular Endothelial Growth Factor (VEGF) levels and increased α-synuclein. Further investigations are warranted to confirm the causal relationship between these observations, disease severity, and progression, as well as their potential role as biomarkers for DLB.

**Supplementary Information:**

The online version contains supplementary material available at 10.1007/s10072-024-07683-6.

## Introduction

Dementia with Lewy bodies (DLB) is the second most common form of neurodegenerative dementia. DLB neuropathological hallmark is Lewy bodies, i.e. intracellular inclusion of alpha-synuclein (α-syn) and phosphorylated alpha-synuclein (p-α-syn) [1]. DLB’s core clinical features are cognitive fluctuation, visual hallucinations, spontaneous parkinsonism, and rapid eye movement behavioral disorder. Despite its prevalence, DLB remains underdiagnosed or misdiagnosed and it is still poorly investigated [2]. Indeed, while in Alzheimer’s disease (AD) extensive research has been done on biomarkers, research on DLB biomarkers is still lacking. Recent studies have suggested that the eye, particularly the retinal changes may represent potential biomarkers for neurodegenerative diseases [3–8]. The eye changes might be especially precious in DLB since beyond the visual hallucinations, part of the core clinical features, clinically it is possible to observe a wide range of visual disorders ranging from visual misperceptions to contrast sensitivity and color perception alterations, ocular motility problems and difficulties in complex visual tasks [9].

The growing interest in studying the eye, particularly the retina, in patients with different kinds of dementia raises from interesting evidence that emerged in previous studies due to several factors [3–8], among others: (1) the concept that the retina is an extension of the brain; (2) the urgent need for the scientific community to have early and accurate biomarkers for diagnosis; (3) the advent of optical coherence tomography (OCT) and optical coherence tomography angiography (OCTA) that provide an opportunity for a non-invasive and quick modality to observe the retinal structures and the retinal vasculature in vivo.

Indeed, OCT and the recently improved implementation of OCT technology with angiographic analysis of the retinal and choroidal vasculature offer a new and more extensive evaluation of the retina and its vasculature in different pathological processes, including neurodegenerative disease. Several studies have reported OCT and OCTA characteristics in Parkinson’s disease (PD) patients with contrasting results, but few have studied the structural OCT changes in DLB and, to the best of our knowledge, just one previous study has investigated retinal microvascular change with OCTA in these patients [10–18].

The purpose of this study is to investigate structural features and microvascular changes in the retina by OCT and OCTA analysis in DLB patients.

## Materials and methods

A cross-sectional cohort study was conducted from April 2022 to June 2023 at the Sapienza University of Rome, Italy. Twenty-three consecutive DLB outpatients from the Department of Human Neurosciences and Mental Health of the Policlinico Umberto I University Hospital of Rome were assessed for eligibility to be enrolled in this study. Parallelly, twenty-eight healthy age and sex-matched patients with no cognitive disorder were consecutively assessed for recruitment at the Ophthalmology unit of Policlinico Umberto I University Hospital of Rome. DLB diagnosis was made according to the consensus criteria for probable DLB [9]. Inclusion criteria for both groups were: age between 55 and 85 years and written informed consent to participate in the study.

Exclusion criteria for both groups were: history of systemic conditions potentially affecting retinal structures and retinal microvasculature, such as diabetes, uncontrolled hypertension, history of cardiac disease, and other neurological disorders (different from DLB), history of severe smoking (> 20 cigarettes/day), alcohol abuse (> 4 drinks/ day for men or > 3 drinks /day for women), history drug consumption with known effects on the retina (e.g., hydroxychloroquine), history of chronic inflammatory systemic diseases, concomitant ocular disease including a history of glaucoma or retinal pathology, history of uveitis, patients with refractive errors > 6 diopters of spherical equivalent, previous intraocular surgery other than cataract surgery, and low quality or unreliable OCT and OCTA images. The healthy controls (HC) had to obtain a Mini-Mental State Examination (MMSE) score > 27.

### Clinical evaluation

All patients underwent physical examinations, and standard laboratory tests at the time of enrollment and they had to have an MRI scan compatible with DLB diagnosis and excluding secondary causes of dementia, such as cerebral vasculopathy. All patients underwent a neurological examination and a structured clinical interview with caregivers to assess the presence of at least two of the core DLB clinical features: parkinsonism (i.e. hypokinesia, rest tremor, postural instability, rigidity), REM (Rapid Eye Movement) sleep behavioral disorders, cognitive fluctuations, and visual hallucinations. All the patients underwent a complete neuropsychological evaluation including tests assessing memory, attention, language, praxis, and executive functions.

All subjects underwent the Mini-Mental State Examination (MMSE) to measure global cognitive decline [19, 20]. In patients, we also assessed the Activities of Daily Living (ADL) and Instrumental Activities of Daily Living (IADL) to evaluate the functional impairment [21]. Patients underwent an extensive neuropsychological evaluation including: (1) Rey’s Auditory Verbal Learning Test and Rey-Osterrieth Complex Figure Test [22], and Babcock Story Recall Test [23] for the episodic memory domain; (2) Digit Span and Corsi Block-Tapping Test for short term memory and working memory domain [24]; (3) the Visual Search Test [23] and Trail-Making Test (part A) [25] for the attentional domain; (4) the Clock Drawing Test [26] and Copy of the Rey-Osterrieth Complex Figure Test [22] for visuo-constructional skill assessment; (5) Boston Naming Test [27, 28] for language; (6) the Frontal Assessment Battery [29], Raven’s Colored Progressive Matrices (RCPM) [30], Trail-Making Test (part B and B-A) [25] and Semantic [31] and Phonemic Verbal Fluency Test [22] for executive functions; (7) Visual Object and Space Perception battery (VOSP) [32], the Benton lines test [33] to assess early visual processing abilities and complex visuo-perceptual functions.

### Ophthalmological evaluation

All subjects underwent a complete ophthalmological evaluation, including visual acuity measured by Early Treatment Diabetic Retinopathy Study (ETDRS) chart, slit lamp biomicroscopy, IOP measurement, indirect fundoscopic examination, optical biometry (IOL Master 500, Carl Zeiss Meditec, Dublin, CA), spectral domain (SD) OCT and OCTA imaging. All procedures adhered to the tenets of the Declaration of Helsinki and the Ethics Committee of the Sapienza University of Rome approved the study.

### SD-OCT

Structural SD-OCT (Spectralis Family Acquisition Module, V 6.0.11.0 Heidelberg Engineering Heidelberg, Germany) was performed with a raster 20°x 20°, 19-line-scan protocol with an interval between scans of 240 nm and at least 9 frames averaged for each scan. The automated segmentation protocol (Heidelberg Eye Explorer V1.9.10.0, Heidelberg, Germany) was used to segment the retinal layers and measure the Central Macular Thickness (CMT) and the following retinal layers thicknesses: Retinal Nerve Fiber Layer (RNFL), Ganglion Cell Layer- Inner Plexiform Layer complex (GCL-IPL), Inner Nuclear Layer (INL), Outer Plexiform Layer (OPL), Outer Nuclear Layer (ONL), Retinal Pigment epithelium (RPE), Inner Retinal Layer (IRL), Outer Retinal Layer (ORL) [34]. Two experienced investigators (M.G., G.M.A.) evaluated the automated segmentation and manually corrected for any misalignment. The average measurements were recorded within the 1-mm area of the ETDRS macular grid. The RNFL and GCL-IPL thicknesses were further measured in the inner ring of the ETDRS macular grid. Patients whose images had inadequate signal strength (< 20) were excluded.

### OCTA

RTVue XR Avanti Spectral-Domain OCT System (Optovue, Inc., Fremont, CA, USA) equipped with AngioVue software (Version 2015.1.0.90) was used to measure retinal and choroidal microvasculature, quantifying the flow area, nonflow area, and Vessel Density (VD). The Motion Correction Technology function was adopted to ensure that the eye movement was automatically correct. The scan speed was 70,000 A-scans per second and the scan area, centered on the fovea was 3 × 3 mm2. The acquisitions were performed by orthogonal registration and merging of two consecutive scans. The size of the exported OCT images was 304 × 304 pixels. The Superficial Capillary Plexus (SCP) was automatically segmented between 3 μm below the Internal Limiting Membrane (ILM) and 15 μm below the IPL. The Deep Capillary Plexus (DCP) consisted of the capillaries in the area between 15 μm below the IPL and 70 μm below the IPL. The automated segmentation was checked before data analysis. In both of that, we evaluated the vessel density, respectively defined as Superficial Vessel Density (SVD) and Deep Vessel Density (DVD). These were defined as the percentage of area occupied by vessel lumen following the binary reconstruction of images. The percentage of vessels was defined in the ETDRS chart where the fovea center was automatically determined and the inner and outer diameters of the parafoveal area were 1 and 2.5 mm respectively. The SVD and DVD were measured in the whole macula images (WMI), in the fovea, and in the parafovea area. Automated measurement of the Foveal Avascular Zone (FAZ) area was obtained using the nonflow area measurement option. An avascular area defined by automatic border detection was quantified. We also measured the flow area. This is a measurement that quantifies the area of flow signal within a predefined outer retinal (defined as OPL + 10 m to BRM-10 m) or choriocapillaris slab (defined as BRM-10 m to BRM + 30 m). Flow detection was performed using the automatic circle area of 3.144 mm2 area centered on the fovea.

All eyes were also scanned using a 4.5 × 4.5 mm scan size centered on the Optic Nerve Head (ONH). The volumetric scan was processed by the same software, and we recorded the automated vessel density measurements from the inside disc and peripapillary area. After the automated determination of optic disc boundaries, the radial peripapillary capillary (RPC) was automatically segmented from ILM to the outer border of the GCL. The vessel density (VD) of ONH was calculated using the automated algorithm in two concentric circles (1.5 and 3.4 mm in diameter) centered on the disc. The VD of ONH was calculated for the inside of the smaller circle (the inside disk) and the larger circle of the peripapillary areas (ppVD).

The ONH en face image was used to calculate the inside disc RPC vessel density; it was automatically segmented with an inner boundary at the ILM and an outer boundary set at 149 μm below the ILM. Automated measurement of peripapillary RNFL (p-RNFL) thickness in a 3.45 mm ring centered on the optic disc was performed using the same OCTA instrument. Patients whose images had inadequate signal strength (< 7/10) were excluded.

### Statistical analysis

Statistical analysis was performed using STATA, v. 18.0 (2023, StataCorp, TX, USA). For descriptive statistics, to assess differences between the group of DLB patients and healthy controls, data were analyzed including both eyes of every included patient. Conversely, for inferential statistics, a correction at the patient level was employed to exclude inter-eye correlation from the models. To assess the normal distribution of the continuous variables we used the Shapiro–Wilk test. Variables that were normally distributed were reported as mean ± standard deviation (SD), while those that were not normally distributed were reported as median and interquartile ranges. Parametric and non-parametric values were tested respectively with the unpaired t-test and the Mann–Whitney to verify the differences. Bivariate relationships for parametric values were evaluated by the Pearson coefficient, while Spearman’s rank correlation was employed for nonparametric values. Counts and percentages were employed to report categorical values and Fisher’s exact test was used to compare them. To identify factors predicting DLB, a logistic regression analysis including age, sex, and factors correlated with diagnosis of DLB in the bivariate analyses was run. In the case of two or more variables with potential collinearity (e.g., whole macular image SVD, SVD of the fovea, and SVD of the parafovea), only one variable was included in the model selecting the one with a stronger correlation with DLB. Factors with *p* < 0.05 were retained as predictors for the disease.

## Results

**Fig. 1:**
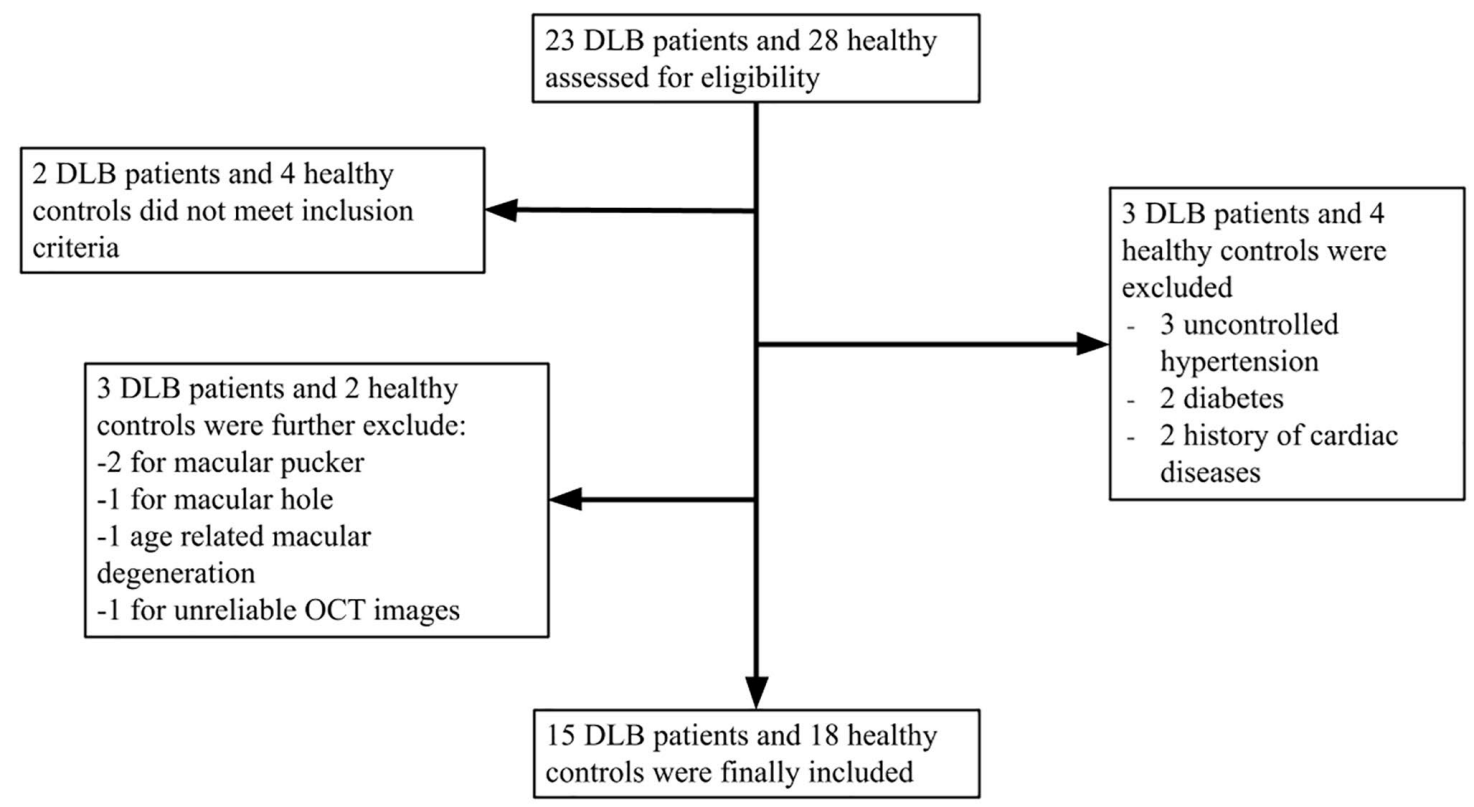
Flowchart outlining the selection process for participants. Initially, 23 individuals with Dementia with Lewy Bodies (DLB) and 28 healthy controls were assessed. After applying inclusion and exclusion criteria, the final cohort included 15 individuals with DLB and 18 healthy controls. OCT = Optical Coherence Tomography

According to the inclusion and exclusion criteria, as shown in Fig. 1, we finally included 15 DLB patients (age 75.9 ± 5.2) and 18 sex and age-matched healthy controls (age 73.6 ± 6.9). Baseline clinical characteristics are described in Table 1. Nine DLB patients had mild small vessel disease qualitatively reported from MRI scan. Overall, no differences were highlighted between the two groups, except for the MMSE (*p* < 0.001). Further, no differences were found regarding the baseline eye characteristics between the two groups as shown in Table 2.

**Table 1:** Population’s characteristics at enrollment: differences between healthy controls and dementia with Lewy bodies (DLB) patients

Characteristics	Healthy Controls (*n* = 18)	DLB patients (*n* = 15)	*p*-value
Age, mean ± SD	73.6 ± 6.9	75.9 ± 5.2	0.295*
Sex (female)	9 (50.0%)	5 (33.3%)	0.271‡
MMSE, (median, IQR)	29 (29; 30)	21 (19; 27)	***< 0.001***†
Previous cataract surgery, n (%)	6 (33.3%)	3 (20.0%)	1.000‡

*Unpaired t-test, †Mann-Whitney U test, ‡Fisher exact test

*P*-values < 0.05 are given in bold-italic entries

MMSE: Mini-mental state examination

**Table 2:** Eyes’ characteristics at enrollment: differences between healthy eyes and dementia with Lewy bodies (DLB) eyes

Characteristics	Total eyes (*n* = 66)	Control eyes (*n* = 36)	DLB eyes (*n* = 30)	*p*-value
LogMAR, (median, IQR)	0 (0; 0.05)	0 (0; 0.05)	0.02 (0; 0.05)	0.145†
SEQ, D (median, IQR)	0 (-1; 1)	0.31 (-0.82; 0.25)	0 (-1; 1)	0.581†
ACD, mm (mean ± SD)	3.32 ± 0.51	3.22 ± 0.49	3.45 ± 0.50	0.070*
AXL, cm (mean ± SD)	23.62 ± 0.81	23.46 ± 0.98	23.83 ± 0.49	0.071*
IOP, mmHg (mean ± SD)	14.3 ± 1.8	14.7 ± 1.8	13.9 ± 1.8	0.073*

*Unpaired t-test, †Mann-Whitney U test

BCVA: Best Corrected Visual Acuity; LogMAR: Logarithm of the Minimum Angle of Resolution; SEQ: spherical equivalent; D: diopters; ACD: anterior chamber depth; AXL: axial length; IOP: intraocular pressure

### SD-OCT measurements

When comparing the SD-OCT measurements with control subjects, the CMT of DLB patients was reduced (*p* < 0.001). As shown in Table 3, the 3 mm NFL was thinner in patients compared to controls (*p* = 0.036), and similarly, the thickness of GCL-IPL complex was reduced in patients both at 1 mm (*p* = 0.003) and 3 mm (*p* = 0.003). Additionally, within the DLB group, there was a significant reduction in the IRL (*p* = 0.001).

**Table 3:** Optical coherence tomography (OCT) measurements of enrolled eyes: differences between healthy eyes and dementia with Lewy Bodies (DLB) eyes

OCT measurements (mean ± SD or median and IQR)	Total (*n* = 66)	Control eyes (*n* = 36)	DLB eyes (*n* = 30)	*p*-value
CMT, µm	271 (257; 286)	284.5 (262; 290)	263.5 (252; 271)	***< 0.001***†
NFL, µm (1 mm)	13.1 ± 2.0	13.4 ± 2.2	12.8 ± 1.7	0.185*
NFL, µm (3 mm)	23.0 ± 2.7	23.7 ± 2.9	22.3 ± 2.2	0.036*
GCL + IPL, µm (1 mm)	34.8 ± 6.2	36.8 ± 7.2	32.3 ± 3.6	0.003*
GCL + IPL, µm (3 mm)	83.8 ± 11.6	87.5 ± 8.5	79.2 ± 13.2	0.003*
INL, µm	22.7 ± 6.3	22.4 ± 6.0	23.0 ± 6.7	0.681*
OPL, µm	26.5 (25; 31)	29 (25; 31.5)	26 (25; 29)	0.067†
ONL, µm	87.9 ± 9.6	89.9 ± 9.6	85.6 ± 9.1	0.070*
RPE, µm	16.7 ± 2.3	17.1 ± 2.3	16.1 ± 2.2	0.074*
IRL, µm	183.1 ± 19.8	190.1 ± 20.0	174.8 ± 16.1	0.001*
ORL, µm	86.5 ± 5.5	86.8 ± 6.1	86.2 ± 4.7	0.643*

*Unpaired t-test, †Mann-Whitney U test

*P*-values < 0.05 are given in bold-italic entries

CMT: Central Macular Thickness; NFL: Nerve Fiber Layer; GCL-IPL: Ganglion Cell Layer-Inner Plexiform Layer complex: INL: Inner Nuclear Layer, OPL: Outer Plexiform Layer, ONL: Outer Nuclear Layer, RPE: Retinal Pigment epithelium, IRL: Inner Retinal Layer, ORL: Outer Retinal Layer

### OCTA measurements

Results from OCTA analysis are shown in Table 4. Compared to controls, SVD in the whole macula image was reduced in DLB subjects (*p* = 0.007). Similarly, SVD was reduced also at the foveal and parafoveal areas (*p* = 0.039 and 0.038, respectively). Further, regarding DVD, a reduction in density was found in the whole image (*p* = 0.004) and, parallelly, at foveal and parafoveal levels (*p* = 0.012 and 0.007, respectively). Finally, the FAZ area was found to be higher in the DLB group (*p* = 0.047) as well as the flow in the choriocapillaris (*p* = 0.007).

**Table 4:** Optical coherence tomography angiography (OCTA) measurements of enrolled eyes: differences between healthy eyes and dementia with Lewy Bodies (DLB) eyes

OCT-A measurements (mean ± SD or median and IQR)	Total (*n* = 66)	Control eyes (*n* = 36)	DLB eyes (*n* = 30)	*p*-value
**SVD (%)**				
Whole Image Density (%)	43.5 (39.7; 45.2)	44.5 (42; 47.5)	41.9 (38.2; 44.6)	***0.007†***
Foveal Density (%)	16.0 ± 5.3	17.3 ± 6.2	14.5 ± 3.7	0.039*
Parafoveal Density (%)	46.5 (43.5; 49.2)	47.0 (44.7; 50.6)	46.0 (43.5; 47.9)	***0.038†***
**DVD (%)**				
Whole Image Density (%)	49.3 ± 4.0	50.6 ± 4.2	47.8 ± 3.0	0.004*
Foveal Density (%)	32.6 ± 6.1	34.2 ± 7.3	30.5 ± 3.4	0.012*
Parafoveal Density (%)	52.0 ± 3.4	53.0 ± 3.3	50.8 ± 3.1	0.007*
**FAZ area (mm2)**	0.28 ± 0.8	0.26 ± 0.10	0.30 ± 0.05	0.047*
**Flow area (mm2)**				
Outer Retina	0.61 (0.42; 0.89)	0.60 (0.37; 0.82)	0.78 (0.44; 0.93)	0.121†
Choriocapillaris	2.16 (2.08; 2.22)	2.13 (2.05; 2.17)	2.18 (2.15; 2.23)	***0.007***†

*Unpaired t-test, †Mann-Whitney U test

*P*-values < 0.05 are given in bold-italic entries

SVD: superficial vessel density; DVD: deep vessel density; FAZ: foveal avascular zone

In Table 5 results of OCTA of the optic disk are shown. Optic Nerve Head (ONH) analysis showed a reduction in the peripapillary vessel density (ppVD, *p* = 0.013). Further, compared to controls, DLB patients showed a reduction in the total p-RNFL (*p* = 0.001), and both in the superior and inferior subfields.

**Table 5:** Optical coherence tomography angiography (OCTA) measurements of the optic disc of enrolled eyes: differences between healthy eyes and dementia with Lewy Bodies (DLB) eyes

OCT-A measurements (mean ± SD or median and IQR)	Total (*n* = 66)	Control eyes (*n* = 36)	DLB eyes (*n* = 30)	*p*-value
**HD Angio-Disc**				
Whole Image Density	47.2 ± 3.3	47.8 ± 2.6	46.6 ± 3.9	0.163*
Inside Disc Density	49.6 ± 4.5	49.7 ± 4.5	49.4 ± 4.7	0.730*
Peripapillary Density	49.8 (47; 52.7)	51.4 (47.7; 53.4)	47.9 (45.9; 51.9)	**0.013†**
**ONH**				
Average pRNFL	93 (85; 99)	96 (92; 99)	86.5 (81; 95)	**0.001†**
Superior	93.5 (88; 100)	97 (92; 100)	90 (84; 100)	**0.002†**
Inferior	91 (81; 99)	94 (89; 100.5)	85 (76; 95)	**0.003†**

*Unpaired t-test, †Mann-Whitney U test

*P*-values < 0.05 are given in bold-italic entries

ONH: Optic Nerve Head; pRNFL: peripapillary Retinal Nerve Fibre Layer

### SD-OCT and OCTA biomarkers predicting DLB

**Fig. 2:**
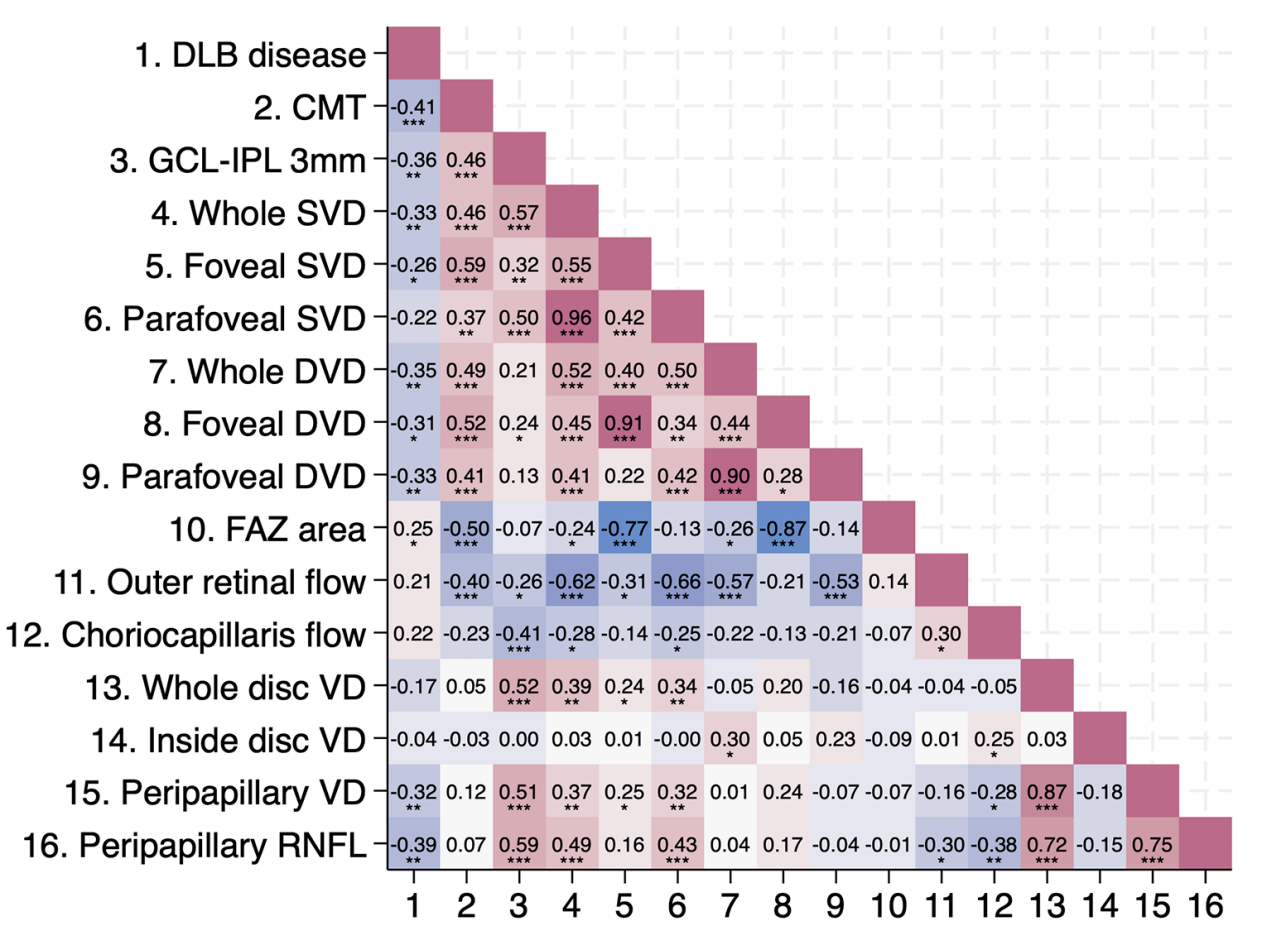
Heat map displaying the correlation coefficients between Dementia with Lewy Bodies (DLB) disease and Optical Coherence Tomography (OCT) Optical Coherence Tomography Angiography (OCTA) parameters. *p < 0.05, **p < 0.01, ***p < 0.001

Figure 2 presents a heatmap of bivariate correlations between DLB disease and SD-OCT/OCTA parameters. DLB disease inversely correlates with CMT (*p* = 0.001), GCL-IPL complex at 3 mm (*p* = 0.003), ppVD, and p-RNFL, along with all SVD and DVD parameters, except for parafoveal SVD. Conversely, DLB shows a direct correlation with FAZ area (*p* = 0.047). Overall, no correlations were observed between SD-OCT and OCTA parameters across the two fellow eyes at the patient level. No significant correlations were found between neuropsychological test scores and OCTA values.

To find potential markers for predicting DLB, we conducted a multivariate logistic regression analysis, correcting for age and sex. We also adjusted for similarities between measurements from both eyes of an individual to avoid bias. As a result, whole SVD, whole DVD, ppVD, and GCL-IPL thickness at 3 mm were retained in the analysis. The outcome of the logistic regression (cons. 25.45, CI 1.78 to 49.11, R2 = 0.26) indicated reduced ppVD as a potential predictive factor for the disease, with a coefficient of -0.18 (CI -0.34 to -0.02, *p* = 0.030).

## Discussion

In this study, we investigated structural and microvascular changes in the retina of DLB patients compared to healthy subjects. Our primary objective was to detect retinal changes and eventual pathological findings; secondarily, we surveyed potential markers that could serve as indicative of the disease. Our results showed that both superficial and deep vessel densities in the whole macula images (WMIs), at the foveal and parafoveal levels, as well as peripapillary vessel density (ppVD) were decreased in patients with DLB. Moreover, we found a reduction in CMT and specifically a thinning of the GCL-IPL complex in DLB subjects compared to healthy controls. In the DLB group, we also identified a reduction in p-RNFL thickness in both the superior and inferior subfields. Following logistic regression analysis, ppVD was retained as a good predictor for diagnosis of DLB among all the evaluated factors.

As previously shown in other neurodegenerative diseases, our data suggest that retinal capillary impairment also occurs in DLB as enhanced by the correlation with lower SVD and DVD [5–7, 11]. This evidence may mirror in the eye the hypoperfusion observed in DLB patients especially in parieto-occipital area [3, 35–38] functionally implicated in the high-order visual processing. From a biological point of view, the decreasing of vessel density might be explained by a reduction of capillary density due to a reduced level of Vascular Endothelial Growth Factor (VEGF). This hypothesis is supported by a post-mortem study showing lower VEGF levels and reduced capillary density in the occipital cortex of a well-characterized cohort of DLB patients, suggesting that lower micro-vessel density is responsible for lower occipital blood flow in DLB [39]. Along with impaired levels of VEGF, significantly lower levels of factor VIII-related antigen, a marker of capillary density, and increased levels of α-synuclein (α-syn) and phosphorylated α-synuclein (p-α-syn) have been demonstrated. These findings suggest that the loss of micro-vessels may result from VEGF deficiency, secondary to the accumulation of α-syn and p-α-syn. These results are supported by other studies where the overexpression of p-α-syn is shown to trigger VEGF decline [39, 40]. In this way, a vicious cycle could set in: the accumulation of p-α-syn leads to cerebral ischemia and VEGF decline, which still further promotes the accumulation of p-α-syn, accelerating the progression of the decrease. A similar mechanism could explain the retinal vascular and structural alterations that we observed on OCTA. A previous study identified the presence of p-α-syn–reactive cells in the retina of patients with DLB, as well as in PD patients [41]. In addition, they established that p-α-syn was present in the retinal ganglion cells. According to these observations, we may speculate that the molecular mechanism leading to the loss of neuronal cells starts from the anomalous accumulation of p-α-syn that in turn promotes VEGF decline, retinal capillary loss, and retinal atrophy. Indeed, consistent with previous studies [13–15], our results showed a reduction of the central macular retinal thickness, specifically of the GCL-IPL complex in DLB patients, probably as a result of the SVD and DVD reduction.

In the current study, we observed that lower ppVD was associated with DLB diagnosis, and further, it was retained as a predictive factor. Our results are different from findings reported in a single previous study analyzing DLB patients with OCTA [18]. Thus, Joseph et al. reported an increase in ppVD compared to a control group. This discrepancy with our findings may be attributed to methodological differences, as the previous study did not include measurements of axial length (AL) or assessment of intraocular pressure (IOP), both of which are known to significantly influence OCTA values. Another possible explanation for the difference in our results might be due to the degree of cognitive impairment, since the DLB patients included in the previous study were less impaired than patients included in our study. It is possible that the increased angiogenesis is a compensatory mechanism occurring in the early disease stage, and then lost as the disease progresses [42]. Further studies involving larger cohorts including DLB patients in different disease stages are needed to fully understand vascular and structural retinal changes.

Another interesting finding is the p-RNFL reduction in DLB patients. In the literature, decreased p-RNFL thickness was associated with several central neurodegenerative diseases, specifically AD [5–7] and PD [11–14]. Our results reflect those of Moreno-Ramos and colleagues, who also found p-RNFL thickness reduction in DLB patients and a correlation with patients’ MMSE and Mattis Dementia Rating Scale scores [17]. Additionally, Price and colleagues (2016) demonstrated the anomalous accumulation of enhanced Green Fluorescent Protein-α-syn (eGFP-α-syn) in retinal images of a transgenic mouse model of DLB/PD, with a higher distribution in the peripapillary region. In particular, the immunocytochemical analysis localized the eGFP-α-syn in the retinal ganglion cells [43, 44]. Furthermore, the presence of p-α-syn immunoreactive nerve fibers has been observed in one DLB patient with an orientation that was radial to the optic disc [45]. Finally, previously described alterations in glutamate receptor expression and signaling in DLB could contribute to p-RNFL thinning [46, 47] Specifically, dysfunction in retinal glutamate neurotransmission, particularly involving N-methyl-D-aspartate (NMDA) receptors, has been implicated in various diseases affecting the optic nerve and nerve fiber layers [48, 49]. These observations may support the hypothesis of an involvement of the peripapillary zone in DLB, which could explain the alterations of vessel density and thickness found in our study.

This study has some limitations. The major limitation is the small sample size which did not allow us to make broad conclusions about these results nor to investigate the association with the severity of DLB core symptoms. However, our study provides preliminary data that warrants a larger study. Furthermore, our study design lacked a comparative analysis with other neurodegenerative diseases, such as AD and PD, which could provide valuable insights into the retinal alterations observed in DLB patients. This comparison across multiple neurodegenerative conditions would enhance our understanding of the distinct retinal changes associated with DLB and facilitate the identification of novel retinal biomarkers for the disease. Future studies with larger sample sizes and including AD and PD groups are warranted. Moreover, in this study, it was not possible to quantify the extent of small vascular disease from MRI, nor was it possible to perform correlations with OCT and OCTA values. Future studies including these MRI measures will help clarify whether a relationship exists between vascular retinal changes and cerebral small vessel alterations. Among the strengths of this research, it should be mentioned that we used a strict protocol of inclusion and exclusion criteria, age and sex-matched population for healthy controls, and the employment of correction for potential inter-eye correlation in the regression analyses.

## Conclusions

Our study showed retinal alterations in DLB patients, including OCT structural changes such as thinning of the GCL-IPL complex and p-RNFL, as well as OCTA microvascular changes characterized by reductions in SVD and DVD percentages, and especially ppVD values. These findings, while preliminary and requiring cautious interpretation, indicate that both structural and vascular retinal changes could serve as valuable biomarkers for DLB.

Given the close relationship between the eye and the brain, their shared embryonic origin, and the non-invasive nature of ocular screening, particularly retinal examination, OCT and OCTA metrics are promising candidates for identifying novel biomarkers. Early and accurate diagnosis is crucial for enabling timely treatment interventions, and establishing these ocular biomarkers could greatly enhance the clinical management of DLB, improving patient outcomes and quality of life.

## Electronic supplementary material

Below is the link to the electronic supplementary material.

Supplementary Material 1

## Data Availability

The data presented in this study are available on reasonable request from the corresponding author, after board approval. The data are not publicly available due to informed consent restrictions that aim to protect the privacy of research participants.
