# Supplementary material for: Neuroretinal and microvascular retinal features in dementia with Lewy body assessed by optical coherence tomography angiography
Source: Neurol Sci. 2024 Aug 17;46(1):185–94. doi: 10.1007/s10072-024-07683-6 (PMC11698750; doi:10.1007/s10072-024-07683-6)
Supplement: Supplementary file 1 — Supplementary Material 1 [file 10072_2024_7683_MOESM1_ESM.docx]

|  | DLB (n=15) |
| --- | --- |
| RAVLT immediate | 17,3±10,9 |
| RAVLT delayed | 2,9±3,3 |
| Prose memory IR | 2,8±2,5 |
| Prose memory DR | 1,8±2,2 |
| Prose memory total | 2,5±3,7 |
| RCFT IR | 6,8±7,1 |
| RCFT DR | 1,1±1,7 |
| DS forward | 4,8±0,9 |
| DS backward | 3,1±0,9 |
| CBT forward | 3,3±0,9 |
| CBT backward | 2,8±1,8 |
| VS | 29,8±13 |
| TMT-A | 189,8±96,1 |
| TMT-B | 144,6±156,8 |
| FAB | 9±4 |
| RCPM | 15,2±8,1 |
| RCFT copy | 10,2±7,3 |
| CDT FD | 7,2±3,2 |
| CDT PD | 5,4±4 |
| CDT ED | 17±9,7 |
| CDT TOT | 29,6±15 |
| PVF | 19,6±10 |
| SVF | 21,1±11,1 |
| BNT | 22,6±11 |
| VOSP visuoperceptual. | 0,6±0,1 |
| VOSP visuospatial | 0,7±0,1 |
| Benton | 14,1±4,7 |

Supplementary Table 1: Neuropsychological test scores (mean ± standard deviation)

Notes: IR: immediate recall, DR delayed recall, RAVLT Rey's auditory verbal learning test, DS Digit span, CBT Corsi block tapping test, RCFT Rey‐Osterrieth complex figure test, VS visual search test, TMT‐A trail making test part A, TMT‐B trail‐making test part B, PVF phonemic verbal fluency, SVF semantic verbal fluency, BNT boston naming test, CDT clock drawing test, FD free drawing condition, PD pre-drawn condition, ED examiner-drawn condition, RCPM. Raven's colored progressive matrices; FAB frontal assessment battery
